# Associations of gut bacterial classes Clostridia and Deltaproteobacteria with type 2 diabetes and Alzheimer’s disease: A two-sample Mendelian randomization study

**DOI:** 10.1097/MD.0000000000048685

**Published:** 2026-05-15

**Authors:** Xueying Gao, Lingling Wang

**Affiliations:** a Department of Postgraduate, School of Clinical Medicine, Beihua University, Jilin, China; b Stroke Unit, Affiliated Hospital of Beihua University, Jilin, China.

**Keywords:** Alzheimer disease, bacterial class, causality, *Clostridia*, *Deltaproteobacteria*, gut microbiota, Mendelian randomization, type 2 diabetes

## Abstract

Using a 2-sample Mendelian randomization (MR) approach, we evaluated MR evidence for genetically proxied effects of 10 pre-specified gut bacterial classes on type 2 diabetes mellitus (T2DM) and Alzheimer disease (AD), and assessed MR evidence for a direct genetically proxied effect of T2DM on AD. Mediation by T2DM was explored where feasible. We performed 2-sample MR using MiBioGen gut microbiota GWAS summary statistics as exposure, and DIAMANTE (T2DM) and European AD GWAS data as outcomes. Inverse-variance weighted (IVW) MR was the primary analysis, with MR-Egger, weighted median, and weighted mode as sensitivity analyses. Heterogeneity and directional pleiotropy were assessed, and IVW results were false discovery rate (FDR) corrected using Benjamini–Hochberg. MR analysis using 184 independent SNPs provided no robust evidence for a direct genetically proxied effect of T2DM on AD risk (IVW odds ratio [OR] = 0.98, 95% confidence interval [CI]: 0.94–1.02, *P* = .28). After FDR correction, higher genetically predicted Clostridia abundance was associated with lower AD risk (IVW OR = 0.86, 95% CI: 0.77–0.96, FDR < 0.05), and higher genetically predicted *Deltaproteobacteria* abundance was associated with higher T2DM risk (IVW OR = 1.11, 95% CI: 1.04–1.20, FDR < 0.05). Other classes were not significant after FDR correction. Reverse MR and multivariable MR were not testable due to insufficient SNP overlap after harmonization (effective SNPs < 3). These findings provide MR evidence consistent with genetically proxied effects of *Clostridia* on AD and *Deltaproteobacteria* on T2DM and do not support robust MR evidence for a direct genetically proxied effect of T2DM on AD. Results are hypothesis-generating and require replication in diverse ancestries and follow-up mechanistic/experimental studies, given that included GWAS sources are predominantly of European ancestry.

## 1. Introduction

Alzheimer disease (AD) is the most common neurodegenerative disorder of the central nervous system, characterized by neuronal and synaptic loss, senile plaques, and neurofibrillary tangle (NFT) deposition, exhibiting a progressive onset and course.^[1]^ AD predominantly affects individuals over 65 years of age. Early manifestations include memory loss and mild cognitive decline; as the disease progresses, it can impair language, executive function, and activities of daily living, severely affecting patients’ quality of life and imposing a heavy social and economic burden.^[2]^ There is a close and complex association between type 2 diabetes mellitus (T2DM) and AD. Epidemiological studies suggest that patients with T2DM are at a significantly higher risk of cognitive decline and dementia.^[3,4]^ The 2 conditions share common pathophysiological foundations, including insulin resistance, chronic inflammatory responses, oxidative stress, insulin-like growth factor (IGF) signaling pathway abnormalities, glycogen synthase kinase-3β (GSK-3β) activation, and the formation of amyloid-beta (Aβ) and NFTs.^[4,5]^ Consequently, AD has been hypothesized to be “type 3 diabetes.”^[5]^ However, recent studies using 2-sample Mendelian randomization (MR) to specifically assess the causal relationship between T2DM and AD have yielded inconsistent results; some support an increased risk of AD associated with T2DM, while others fail to observe a significant causal effect.^[6–9]^ Due to differences in data sources, instrumental variable selection, and methodology, a unified conclusion regarding whether T2DM is a direct causal risk factor for AD remains elusive.

In addition to metabolic pathways involving T2DM, the development of AD is also influenced by other biological systems. In recent years, the microbiota–gut–brain axis theory has developed rapidly.^[10]^ Substantial basic and clinical evidence suggests that the gut microbiota, as a critical physiological regulatory system, can influence the central nervous system through neural, immune, and endocrine pathways.^[11–13]^ Gut dysbiosis, characterized by reduced microbial diversity and remodeling of dominant flora, has been demonstrated in patients with AD and mild cognitive impairment.^[14–19]^ Microbial metabolites (e.g., short-chain fatty acids, lipopolysaccharides, bile acids, and amino acid metabolites) are thought to be involved in key pathological processes such as neuroinflammation, Aβ deposition, and altered synaptic plasticity.^[15–17,20]^ Conversely, patients with T2DM also exhibit significant alterations in the structure and function of the gut microbiota, which are closely associated with glucose tolerance, insulin resistance, and diabetic complications.^[21,22]^ These findings suggest that the gut microbiota may play a common upstream or partially mediating role in the “metabolic abnormality–neurodegenerative disease” pathological chain.

Conventional observational studies are susceptible to confounding factors such as lifestyle, medication use, comorbid cardiovascular disease, and socioeconomic status. Furthermore, distinguishing between causality and comorbidity is difficult, leading to uncertainty in conclusions. Mendelian randomization utilizes genetic variants associated with the exposure as instrumental variables. Because genotypes are randomly assigned at conception and are relatively independent of acquired environmental factors, MR can mimic randomized controlled trials to some extent, allowing for causal inference between exposure and outcome.^[23]^ Compared with conventional cohort or case–control studies, MR offers advantages in reducing confounding bias, minimizing reverse causation, and improving the credibility of causal inference. It has been widely applied in studies investigating the causal relationships between metabolic and neurological diseases.^[23,24]^

Although studies have explored the associations between T2DM, AD, and gut microbiota from various perspectives, several key issues remain unresolved. First, previous MR studies have mostly examined the “T2DM–AD” or “gut microbiota–AD/T2DM” relationships separately, lacking a systematic evaluation of the causal network among the 3 within a unified genetic framework. Second, most gut microbiota MR studies employ a genome-wide scanning strategy, yielding scattered positive results with limited reproducibility and failing to establish stable evidence for specific bacterial classes or key flora. Third, hypotheses regarding whether the “AD–T2DM relationship is independent of gut microbiota” or whether “specific microbiota mediate AD risk through T2DM” lack systematic validation based on large-scale GWAS data.

Against this background, this study integrates GWAS summary statistics from the MiBioGen Consortium (gut microbiota), the DIAMANTE Consortium (T2DM), and a large-scale European AD GWAS. Using a 2-sample MR design, we aim to answer the following questions at the genetic level: Is there MR evidence for a direct genetically proxied effect of T2DM on AD? Among 10 pre-specified gut bacterial classes supported by prior literature and clinical evidence, which ones show consistent MR evidence in relation to T2DM or AD and can be considered key nodes of the gut–metabolism–brain axis? Among the identified key bacterial classes, can mediation by T2DM be assessed given SNP overlap after harmonization and instrument feasibility? By systematically analyzing these questions, we aim to delineate the genetic evidence linking gut microbiota, T2DM, and AD, prioritizing candidates for downstream replication and mechanistic/experimental studies centered on specific microbiota.

## 2. Methods

### 2.1. Research design and data sources

**Figure 1.**
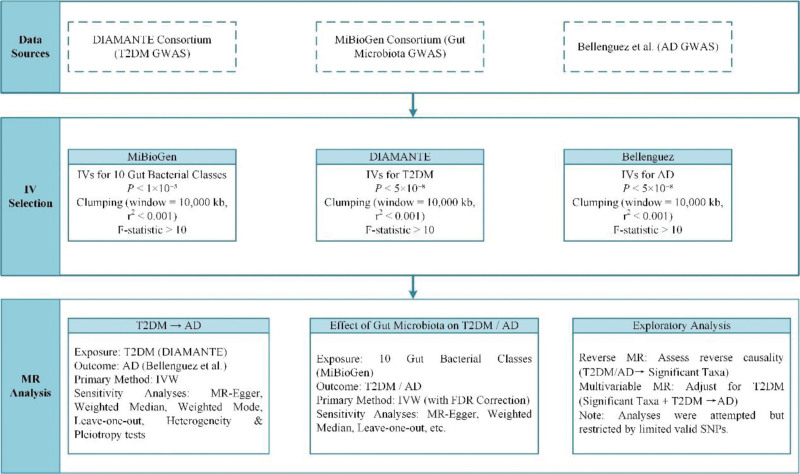
Flowchart of research design and analysis.

This study utilized a 2-sample MR approach based on publicly available GWAS summary statistics to assess MR evidence for genetically proxied effects among gut bacterial classes, T2DM, and AD. The overall analysis workflow is shown in Figure. 1. First, T2DM genetic instrumental variables were constructed using the DIAMANTE Consortium European T2DM GWAS to assess MR evidence for a genetically proxied effect of T2DM on AD. Second, 10 pre-specified gut bacterial classes were selected from the MiBioGen Consortium gut microbiota GWAS. Their genetically predicted relative abundances were used as exposures in univariate MR analyses with T2DM and AD as outcomes, respectively, to screen for classes significantly associated with metabolic or neurodegenerative diseases. Finally, for bacterial classes that remained statistically significant after multiple testing correction, reverse MR and multivariable MR (MVMR) were attempted to explore whether T2DM plays a mediating role in the “gut microbiota–AD” pathway, subject to technical feasibility.

Three sets of publicly available GWAS summary statistics were included in this study. The baseline characteristics are shown in Table 1.

**Table 1:** Characteristics of included GWAS datasets.

Dataset	Phenotype/Use	Study design	Sample size	Ancestry
MiBioGen Consortium	Gut bacterial class relative abundance (exposure)	Multi-cohort GWAS based on 16S rRNA sequencing of fecal samples	18,340 participants from 24 cohorts	Multi-ethnic, predominantly European
DIAMANTE Consortium	T2DM (Exposure/Outcome)	Case–control GWAS meta-analysis	74,124 T2DM cases; 824,006 controls	European
Bellenguez et al.	AD (Outcome)	Two-stage case–control and proxy-case GWAS meta-analysis	111,326 AD/ proxy-AD cases; 677,663 controls	Predominantly European

AD = Alzheimer’s disease, GWAS = genome-wide association study, SNP = single-nucleotide polymorphism, T2DM = type 2 diabetes mellitus.

#### 2.1.1. Gut microbiota data

Gut microbiota GWAS data were obtained from the MiBioGen Consortium, which performed 16S rRNA sequencing on fecal samples from 18,340 subjects across 24 cohorts (predominantly of European ancestry). Relative abundance phenotypes at different taxonomic levels (phylum, class, order, family, and genus) were obtained using a unified bioinformatics pipeline, followed by genome-wide association analysis. This study selected GWAS summary statistics at the Class level.

#### 2.1.2. Type 2 diabetes data

T2DM exposure and outcome data were obtained from the DIAMANTE Consortium GWAS for European populations. This study employed a case-control design, integrating data from multiple cohorts, and included approximately 74,124 T2DM cases and 824,006 controls (all European ancestry). The data provided effect estimates (β), standard errors (SE), *P*-values, and allele frequencies for SNPs.

#### 2.1.3. Alzheimer disease data

AD outcome data were derived from the multicenter AD/dementia GWAS summary statistics published by Bellenguez et al. The study sample was primarily European, including 111,326 clinically diagnosed AD or AD-like dementia cases and 677,663 controls. Some cases and controls were inferred based on UK Biobank family history information.

All included GWAS studies were approved by the respective ethics committees, and informed consent was obtained from subjects. This study used only de-identified publicly available summary statistics and did not involve individual-level data; thus, separate ethical approval was exempted.

### 2.2. Exposure and outcome definitions

This study defined 2 categories of exposures and 2 primary outcomes: Exposure 1: The relative abundances of 10 pre-specified gut bacterial classes were selected from the MiBioGen Consortium database as exposure phenotypes. These included: *Actinobacteria*, *Alphaproteobacteria*, *Bacilli*, *Bacteroidia*, *Betaproteobacteria, Clostridia*, *Coriobacteriia*, *Deltaproteobacteria*, *Erysipelotrichia*, and *Gammaproteobacteria*. Exposure 2: T2DM genetic instrumental variables were constructed using the DIAMANTE Consortium European T2DM GWAS data to assess MR evidence for a genetically proxied effect of T2DM on AD and to serve as a potential mediator in MVMR. Outcome 1: In the “Gut Bacterial Class → T2DM” analysis, T2DM served as the outcome variable to evaluate MR evidence for genetically proxied effects of each bacterial class on T2DM risk. Outcome 2: In the “T2DM → AD” and “Gut Bacterial Class → AD” analyses, AD served as the outcome variable.

### 2.3. Instrumental variable selection and quality assessment

#### 2.3.1. Construction of instrumental variables for T2DM and AD

The format_data function in the TwoSampleMR package was used to format T2DM and AD GWAS summary data, ensuring uniform SNP coding, allele assignment, and effect direction. Instrumental variable selection criteria were as follows: SNPs reaching the genome-wide significance threshold (*P* < 5 × 10^-8^) in the original GWAS; 2) Clumping was performed to ensure independence, using a 1 Mb physical distance window within the same chromosome, retaining only the SNP with the lowest *P*-value in each window; 3) The F-statistic for each candidate SNP was calculated using the formula *F* = *β*^2^/*SE*^2^. SNPs with *F* > 10 were retained as strong instrumental variables to mitigate weak instrument bias. After screening and clumping, independent instrumental SNPs for T2DM and AD were obtained. Allele harmonization was performed using the harmonise_data function before MR analysis.

#### 2.3.2. Construction of instrumental variables for gut bacterial classes

Given the relatively low heritability of gut microbiota phenotypes, a relaxed screening strategy was adopted for the 10 bacterial classes to balance the number and strength of instrumental variables: The significance threshold was set at *P* < 1 × 10^-5^; Clumping was performed using the same strategy as for T2DM/AD (1 Mb window) to ensure independence; and Candidate SNPs with an F-statistic > 10 were retained. The final number of instrumental variables for the 10 candidate bacterial classes ranged from 9 to 22. After harmonization with T2DM and AD outcomes, the number of effective SNPs ranged from 6 to 18. All retained SNPs had F-statistics > 10, indicating acceptable instrument strength. The number of effective SNPs is shown in Table 2.

**Table 2:** Number of instrumental variables for 10 gut bacterial classes and effective SNPs for each outcome.

Bacterial class	Number of instrumental variables	Number of SNPs for T2DM	Number of SNPs for AD
*Actinobacteria*	20	17	17
*Alphaproteobacteria*	11	8	9
*Bacilli*	22	18	18
*Bacteroidia*	16	16	16
*Betaproteobacteria*	17	13	13
*Clostridia*	17	11	11
*Coriobacteriia*	20	17	17
*Deltaproteobacteria*	13	11	11
*Erysipelotrichia*	13	10	10
*Gammaproteobacteria*	9	6	6

AD = Alzheimer’s disease, SNP = single-nucleotide polymorphism, T2DM = type 2 diabetes mellitus.

### 2.4. MR analysis

#### 2.4.1. Univariate MR analysis and sensitivity assessment

For each exposure–outcome combination, 2-sample univariate MR analysis was performed using the TwoSampleMR package.

#### 2.4.2. Primary analysis

The inverse variance weighted (IVW) fixed-effects model was used as the primary method to estimate MR effects (genetically proxied effects under MR assumptions).

#### 2.4.3. Robustness assessment

Complementary MR methods, including MR-Egger regression, weighted median, and weighted mode, were used as supplementary analyses when their respective assumptions were satisfied, to assess the consistency and robustness of results under different instrumental variable assumptions. The effect estimate β derived from MR analysis represents the change in the log risk of the outcome corresponding to each unit change in the genetic prediction of exposure. For clinical interpretation, β was converted to odds ratios (*OR* = exp(*β*)) and 95% confidence intervals (CI).

#### 2.4.4. Sensitivity diagnostics

To ensure the reliability of MR inference, Cochran Q statistic was used to evaluate heterogeneity in the IVW and MR-Egger models (Q-test *P* < .05 indicates statistical heterogeneity). The MR-Egger intercept term was used to test for the presence of directional pleiotropy (intercept *P* > .05 suggests no evidence of directional pleiotropy; however, pleiotropy cannot be fully excluded). Leave-one-out analysis was performed to evaluate whether the overall MR estimate was driven by any single SNP.

#### 2.4.5. Visualization

Scatter plots, forest plots, funnel plots, and leave-one-out plots were generated to visualize key findings. Scatter plots display the relationship between exposure and outcome effects along with fitted lines; forest plots show individual and combined effect estimates; funnel plots allow visual assessment of pleiotropy; leave-one-out plots reflect changes in the overall effect after excluding individual SNPs.

#### 2.4.6. Multiple testing correction

Given that this study conducted MR analysis on 10 pre-specified gut bacterial classes, the Benjamini–Hochberg method was applied to perform false discovery rate (FDR) correction on the IVW results for the 10 class–outcome combinations to control for Type I errors. An FDR < 0.05 was defined as a statistically significant association, while 0.05 ≤ FDR < 0.10 was considered a marginal association.

#### 2.4.7. Reverse MR and multivariable MR

For bacterial classes that remained statistically significant after FDR correction, reverse MR models were attempted (treating T2DM or AD as exposure and significant bacterial classes as outcome) to evaluate reverse genetic effects. Additionally, multivariable MR (MVMR) was planned to explore the independent effect of bacterial classes on AD after adjusting for T2DM, subject to data availability. If the number of harmonized SNPs available for any reverse MR or MVMR combination was fewer than 3, the model was considered unstable, and quantitative results were not reported; only qualitative descriptions are provided in the discussion.

### 2.5. Statistical analysis

All statistical analyses were performed using R software (version 4.5.2; The R Foundation for Statistical Computing, Vienna, Austria), primarily utilizing the TwoSampleMR, MendelianRandomization, data.table, dplyr, and ggplot2 packages. All tests were 2-tailed, with *P* < .05 considered statistically significant, subject to the aforementioned FDR correction standards.

## 3. Results

### 3.1. MR evidence for the genetically proxied effect of T2DM on AD

Using 184 independent SNPs identified in the DIAMANTE Consortium European GWAS as instrumental variables, univariate MR analysis was conducted to examine MR evidence for a genetically proxied effect of T2DM on AD (Table 3). The IVW model showed no significant MR evidence for a direct genetically proxied effect between T2DM and AD risk (OR = 0.98, 95% CI: 0.94–1.02, *P* = .28). Complementary methods (MR-Egger regression, weighted median, and weighted mode) yielded consistent effect directions with OR values close to 1.00, suggesting limited evidence for a direct genetically proxied effect. Cochran Q statistic indicated no significant heterogeneity, and the MR-Egger intercept was close to zero (*P* > .05), providing no evidence of directional pleiotropy; however, pleiotropy cannot be fully excluded.

**Table 3:** Univariate Mendelian randomization results for T2DM on AD.

Method	SNP count	OR (95% CI)	*P*-value
IVW	184	0.98 (0.94, 1.02)	.281
MR-Egger regression	184	0.93 (0.82, 1.06)	.270
Weighted median	184	0.99 (0.94, 1.04)	.637
Weighted mode	184	0.98 (0.92, 1.05)	.574

CI = confidence interval, IVW = inverse variance weighted, MR = Mendelian randomization, OR = odds ratio, SNP = single-nucleotide polymorphism.

### 3.2. MR evidence for genetically proxied effects of 10 gut bacterial classes on T2DM risk

**Figure 2.**
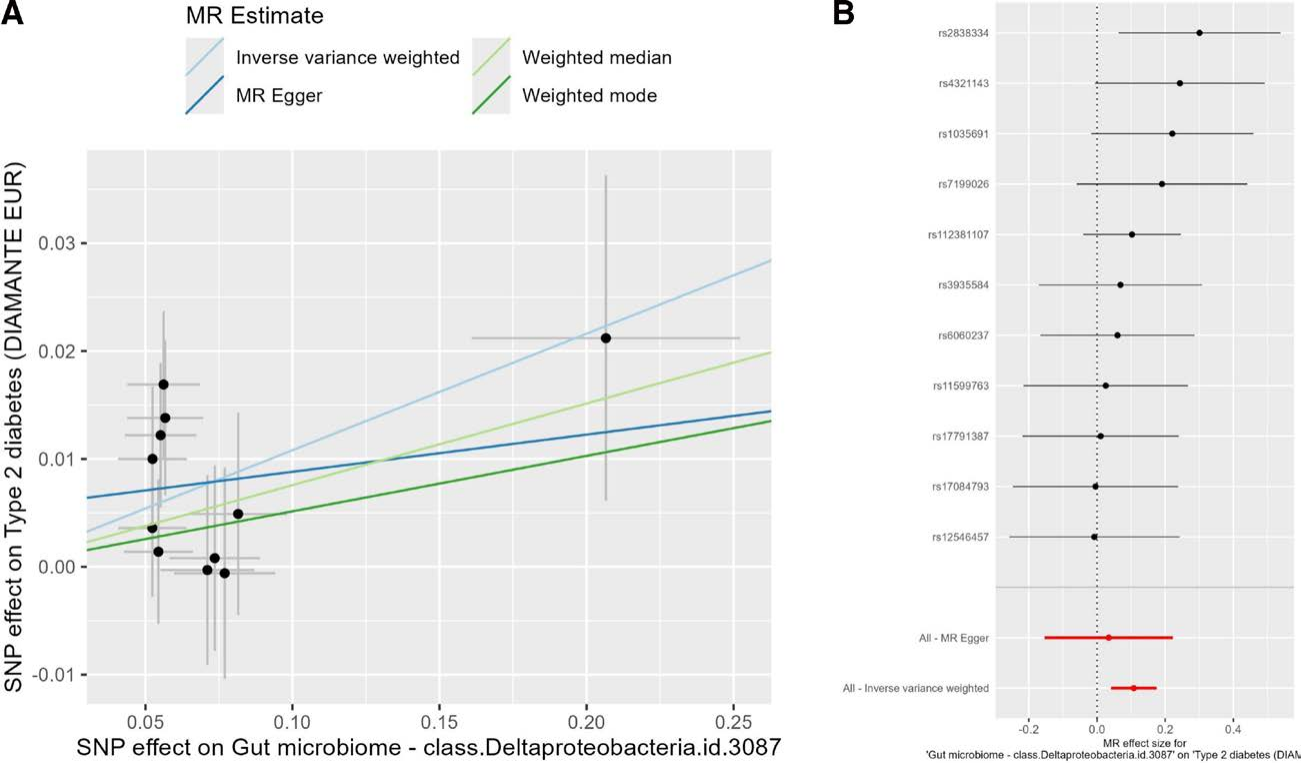
Mendelian randomization results for *Deltaproteobacteria* and T2DM. (A) Scatter plot showing the relationship between SNP effects on *Deltaproteobacteria* and T2DM. (B) Forest plot of single-SNP effects. SNP = single-nucleotide polymorphism, T2DM = type 2 diabetes mellitus.

Univariate MR results for the 10 pre-specified gut bacterial classes and T2DM are shown in Table 4. Overall, most classes showed ORs close to 1.0 and did not reach statistical significance after FDR correction. However, *Deltaproteobacteria* showed a stable risk effect. IVW estimates indicated that a 1-unit increase in the relative abundance of *Deltaproteobacteria* was associated with an approximately 11% higher risk of T2DM (OR = 1.11, 95% CI: 1.04–1.20, *P* = .002, FDR = 0.016). Weighted median and weighted mode methods yielded similar ORs and 95% CIs, with consistent effect directions. As shown in Figure 2, SNPs associated with *Deltaproteobacteria* were predominantly distributed in the upper-right quadrant of the MR scatter plot (Fig. 2A), with the IVW regression line showing a positive slope, reflecting its promoting effect on T2DM. The single-SNP forest plot (Fig. 2B) showed that the Wald ratios and 95% CIs for most loci aligned with the overall risk effect direction, with no single outlier driving the estimate. The funnel plot was generally symmetrical, and leave-one-out analysis showed that excluding any single SNP did not significantly alter the overall effect (Supplemental Digital Content 1.), further supporting the stability and consistency of the MR estimate across sensitivity analyses.

**Table 4:** Mendelian randomization results for 10 pre-specified gut bacterial classes on T2DM risk.

Bacterial class	nIV	Effective SNPs (T2DM)	OR (95% CI)	*P*-value	FDR
*Actinobacteria*	20	17	1.06 (0.99, 1.13)	.083	0.277
*Alphaproteobacteria*	11	8	0.98 (0.89, 1.08)	.719	0.859
*Bacilli*	22	18	1.00 (0.93, 1.07)	.950	0.950
*Bacteroidia*	16	16	0.99 (0.93, 1.05)	.655	0.859
*Betaproteobacteria*	17	13	1.00 (0.93, 1.06)	.969	0.969
*Deltaproteobacteria*	13	11	1.11 (1.04, 1.20)	.002	0.016
*Clostridia*	17	11	0.98 (0.92, 1.05)	.573	0.821
*Coriobacteriia*	20	17	1.06 (0.99, 1.13)	.100	0.277
*Erysipelotrichia*	13	10	1.02 (0.95, 1.10)	.530	0.821
*Gammaproteobacteria*	9	6	0.99 (0.92, 1.07)	.845	0.938

CI = confidence interval, FDR = false discovery rate, IVW = inverse variance weighted, MR = Mendelian randomization, OR = odds ratio, SNP = single-nucleotide polymorphism, T2DM = type 2 diabetes mellitus.

### 3.3. MR evidence for genetically proxied effects of gut bacterial classes on AD risk

**Figure 3.**
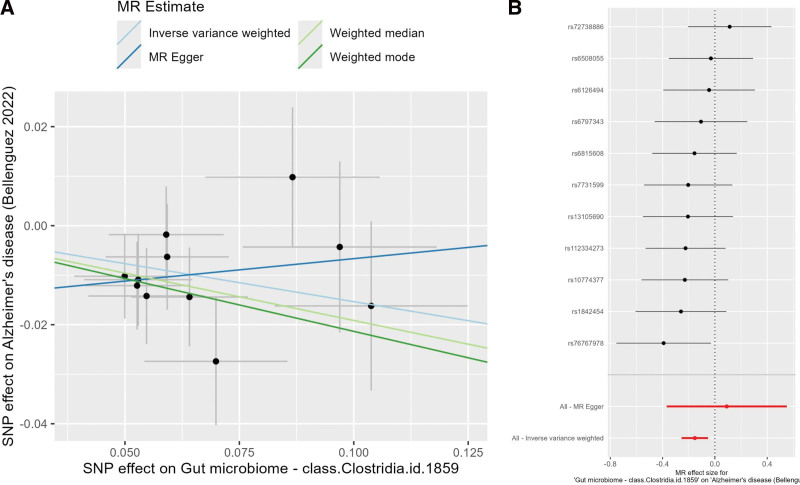
Mendelian randomization results for *Clostridia* and AD. (A) Scatter plot showing the relationship between SNP effects on *Clostridia* and AD. (B) Forest plot of single-SNP effects. AD = Alzheimer disease, SNP = single-nucleotide polymorphism.

Among the 10 bacterial classes, only *Clostridia* exhibited a statistically significant protective effect on AD after FDR correction (Table 5). IVW results indicated that increased relative abundance of *Clostridia* was associated with a reduced risk of AD (OR = 0.86, 95% CI: 0.77–0.96, *P* = .003, FDR = 0.029). The weighted median method produced similar effect sizes, and MR-Egger regression showed a consistent direction, though with slightly wider confidence intervals. Visualization of *Clostridia* and AD results is shown in Figure 3. In the scatter plot (Fig. 3A), the IVW regression line exhibited a negative slope, suggesting an inverse relationship between *Clostridia* abundance and AD risk. The forest plot (Fig. 3B) showed that the Wald ratios and 95% CIs for most SNPs fell within the OR < 1 region, indicating a significant protective effect. Cochran Q test revealed no significant heterogeneity, and the MR-Egger intercept *P*-value was > .05. The funnel plot was generally symmetrical, and leave-one-out analysis showed that excluding any single SNP did not alter the direction of the overall effect (Supplemental Digital Content 2.), suggesting a low likelihood of substantial pleiotropy or single key driver loci. Additionally, *Actinobacteria* showed a nominal *P*-value of approximately .02 for AD but an FDR of 0.11 after correction, indicating only a marginal association; thus, it is not discussed as a primary positive finding.

**Table 5:** Mendelian randomization results for 10 pre-specified gut bacterial classes on AD risk.

Bacterial class	nIV	Effective SNPs (AD)	OR (95% CI)	*P*-value	FDR
*Clostridia*	17	11	0.86 (0.77, 0.96)	.003	0.029
*Actinobacteria*	20	17	1.12 (1.02, 1.24)	.021	0.105
*Alphaproteobacteria*	11	9	0.95 (0.84, 1.07)	.389	0.748
*Bacilli*	22	18	0.98 (0.89, 1.09)	.716	0.895
*Bacteroidia*	16	16	1.02 (0.93, 1.12)	.650	0.895
*Betaproteobacteria*	17	13	1.02 (0.93, 1.11)	.676	0.895
*Coriobacteriia*	20	17	1.08 (0.99, 1.17)	.068	0.227
*Deltaproteobacteria*	13	11	0.93 (0.84, 1.04)	.210	0.525
*Erysipelotrichia*	13	10	0.96 (0.87, 1.06)	.439	0.748
*Gammaproteobacteria*	9	6	1.00 (0.90, 1.12)	.978	0.978

CI = confidence interval, FDR = false discovery rate, OR = odds ratio, SNP = single-nucleotide polymorphism.

### 3.4. Comparative analysis of candidate bacterial classes

Comparing the MR results across the 10 candidate gut bacterial classes for T2DM and AD (Tables 4 and 5) reveals that *Deltaproteobacteria* is the only class significantly positively associated with T2DM risk after FDR correction, while *Clostridia* is the only class significantly negatively associated with AD risk after FDR correction. The remaining classes mostly exhibited neutral or weak effects. These findings suggest that different bacterial classes may participate in distinct pathological pathways along the gut–metabolism–brain axis: *Deltaproteobacteria* appears more relevant to metabolic risk pathways, whereas *Clostridia* appears more relevant to neuroprotective pathways.

### 3.5. Reverse MR and multivariable MR results

We further attempted reverse MR and MVMR analyses for the 2 primary associations described above. However, due to limited overlap between DIAMANTE/Bellenguez instrumental variables and MiBioGen bacterial class GWAS loci, fewer than 3 SNPs remained in the harmonized data after allele harmonization and palindromic SNP removal. This resulted in insufficient model stability to obtain reliable reverse MR estimates. Similarly, in MVMR models with *Clostridia* or *Deltaproteobacteria* and T2DM as joint exposures and AD as the outcome, the effective SNP count was insufficient to support robust MR inference, and therefore quantitative results were not reported. Given these limitations, this study reports only the forward univariate MR analysis results.

## 4. Discussion

### 4.1. Key findings

This study systematically assessed MR evidence for genetically proxied effects among gut microbiota, T2DM, and AD from a genetic epidemiological perspective, utilizing a 2-sample MR method based on pooled data from the MiBioGen Consortium gut microbiota GWAS, the DIAMANTE Consortium T2DM GWAS, and a large-scale AD GWAS.^[24–26]^ The findings yielded 3 key insights. First, MR analysis using the DIAMANTE Consortium T2DM GWAS as exposure and the Bellenguez et al AD GWAS as outcome (incorporating 184 independent SNPs) showed no robust evidence for a direct genetically proxied effect between T2DM and AD risk in the IVW model. Multiple supplementary methods yielded consistent effect directions, with OR values consistently close to 1.00, suggesting limited evidence for a direct genetically proxied effect from T2DM to AD. Heterogeneity and MR-Egger intercept tests also provided no evidence of significant heterogeneity or directional pleiotropy; however, pleiotropy cannot be fully excluded, and the effect estimates were modest and close to the null. Second, among the 10 pre-specified gut bacterial classes, only Deltaproteobacteria (with T2DM) and Clostridia (with AD) remained statistically significant after FDR correction. Increased Deltaproteobacteria abundance was associated with elevated T2DM risk (IVW OR ≈ 1.11), while increased Clostridia abundance was linked to reduced AD risk (IVW OR ≈ 0.86). The effect directions were consistent across different MR methods. Leave-one-out analyses revealed no dominant single SNP, suggesting that at the higher taxonomic level of class, only a few bacterial classes may occupy key positions in the “gut microbiota–metabolism–central nervous system” network. Third, constrained by the limited overlap between T2DM/AD instrumental variables and bacterial class GWAS loci, fewer than 3 SNPs remained available after harmonization for reverse MR and MVMR, resulting in poor model stability. This means that reverse MR and MVMR were not testable due to insufficient SNP overlap after harmonization (effective SNPs < 3), and mediation by T2DM could not be assessed in the present framework. The findings primarily reflect MR evidence consistent with genetically proxied effects of specific classes on T2DM or AD. The complete causal chain among these 3 entities requires further investigation.

### 4.2. Comparison with previous studies and potential mechanisms

Previous epidemiological and experimental studies generally suggest that T2DM and AD share common pathological mechanisms involving insulin signaling abnormalities, chronic low-grade inflammation, oxidative stress, and amyloid metabolism disorders.^[3–5]^ Based on this, some scholars have proposed that AD may represent “a subtype of brain pathology associated with diabetes.”^[5]^ However, most observational studies struggle to adequately control for confounding factors such as lifestyle, comorbidities, and medication use.^[3,4]^ This study, based on large-scale GWAS and utilizing an MR design, mitigated these confounding and reverse causality effects to some extent, yet found no robust evidence for a direct genetically proxied effect from T2DM to AD. Using large-scale GWAS and an MR framework, we observed no robust evidence for a direct genetically proxied effect of T2DM on AD. This null finding should be interpreted as statistically inconclusive rather than mechanistically informative. It may reflect insufficient statistical power to detect small effects, weak-instrument bias, heterogeneity within T2DM phenotypes, and potential survivor/proxy-related biases in AD GWAS. Importantly, absence of evidence is not evidence of absence; therefore, our results do not rule out indirect pathways or shared upstream factors, but they also do not provide direct support for any specific mechanistic route. This aligns with conclusions from long-term follow-up and imaging studies indicating that vascular risk factors play a significant role in diabetes-associated cognitive impairment.^[2–4,9]^ Regarding the gut microbiota, the Deltaproteobacteria class harbors diverse sulfate-reducing bacteria capable of producing metabolites such as hydrogen sulfide and lipopolysaccharides, which exhibit mucotoxic and pro-inflammatory effects. Studies indicate that increased abundance of these bacterial groups is closely associated with heightened intestinal barrier permeability, endotoxemia, and systemic low-grade inflammation.^[13,21,22]^ Chronic inflammation and endotoxin exposure constitute a key pathological basis for the development of insulin resistance and T2DM.^[3,21,22]^ Under conditions controlling for genetic confounding, this study observed an association consistent with a genetically proxied effect between genetically predicted Deltaproteobacteria abundance and T2DM risk, which is directionally consistent with parts of the existing literature. The Clostridia class contains numerous butyrate-producing bacteria. Short-chain fatty acids, particularly butyrate, play crucial roles in maintaining the integrity of the intestinal mucosal barrier, regulating peripheral and central immune responses, suppressing neuroinflammation, and sustaining synaptic plasticity.^[13,15,18,19]^ Animal studies have observed reduced Clostridia abundance and butyrate levels in AD model mice, accompanied by excessive microglial activation and increased amyloid deposition.^[13,15]^ This study observed MR evidence consistent with a genetically proxied effect between genetically predicted increased Clostridia abundance and reduced AD risk, with the effect direction consistent with the aforementioned basic research. However, both Clostridia and Deltaproteobacteria are heterogeneous classes, and class-level GWAS does not resolve species/strain-level functions; inferring specific functional mechanisms (e.g., short-chain fatty acids, lipopolysaccharides, H2S) at the class level is imprecise. Moreover, we did not measure metabolites or inflammatory biomarkers, nor did we perform pathway-specific MR or genetically instrument mechanistic intermediates; therefore, mechanistic interpretations here should be regarded as hypothesis-generating rather than pathway validation. Notably, several studies have utilized datasets such as MiBioGen to conduct large-scale association and MR analyses examining the relationship between gut microbiota and metabolic or neurological disorders, frequently reporting a series of “positive microbiota” at the genus or species level.^[13,15–19]^ However, different studies exhibit significant variations in classification hierarchy, effect direction, and statistical significance. On one hand, this reflects the highly heterogeneous nature of the gut microbiota itself, which is susceptible to environmental and host factors; on the other hand, this also highlights the current limitations of 16S rRNA-based GWAS in terms of sample size, sequencing depth, and taxonomic accuracy. While this study focused on 10 pre-specified bacterial classes, thereby reducing the burden of multiple testing to some extent, it remains challenging to draw definitive conclusions regarding key genera or species at finer taxonomic levels.

### 4.3. Clinical and scientific significance

From a clinical perspective, the findings of this study provide valuable insights into understanding the relationship between T2DM and AD. On one hand, no robust evidence for a direct genetically proxied effect of T2DM on AD was observed at the genetic level. This indicates that when assessing cognitive risk in T2DM patients, T2DM should not be simplistically equated with an inevitable precursor state of AD. Instead, multiple factors should be comprehensively considered, including control of vascular risk factors, coexisting cardiovascular and cerebrovascular diseases, prevention and treatment of diabetic complications, and the impact of long-term medication use.^[3,4,9,20]^ On the other hand, this study suggests relatively stable MR evidence consistent with genetically proxied effects between Deltaproteobacteria and T2DM, as well as between Clostridia and AD. These MR findings do not directly imply that modifying the microbiota through diet, probiotics, or antibiotics will change disease risk. Instead, the primary value of the present work is to prioritize candidate classes for downstream replication, higher-resolution microbial profiling, multi-omics measurements, and mechanistic experiments. Interventional relevance requires dedicated clinical trials and multi-omics evidence linking modifiable exposures to disease outcomes. From the perspective of basic and translational research, this study identified 2 candidate bacterial classes associated with T2DM/AD at the class level. This finding helps narrow the scope of focus within the complex gut microbiota network, providing clearer targets for subsequent experimental studies. Focusing on Deltaproteobacteria and Clostridia, future research can further characterize their representative bacterial species and dominant metabolites, investigate their interactions with host genes and immune pathways, and clarify their specific roles within the “gut microbiota–metabolism–brain” continuum. This will provide an experimental foundation for evaluating biological pathways and translational relevance in future mechanistic and interventional studies.

### 4.4. Advantages and limitations

This study possesses certain advantages. First, the use of pooled GWAS data from large samples and a 2-sample MR design mitigates biases associated with lifestyle, socioeconomic factors, and reverse causality that are common in traditional observational studies.^[23,24]^ Second, by testing predefined hypotheses at the relatively robust taxonomic level of bacterial class and applying FDR correction for multiple comparisons, while integrating multiple MR methods and sensitivity analyses, the reliability of the results was enhanced. At the same time, this study also has several limitations. First, the GWAS sources are predominantly of European ancestry, and microbiome composition varies substantially by ethnicity, diet, and geography; therefore, generalizability and translational interpretation beyond European-ancestry populations are limited and require dedicated replication. Second, microbiome GWAS instruments typically explain limited variance; although we applied F-statistic filtering and sensitivity diagnostics, weak-instrument bias and pleiotropy cannot be fully excluded. Third, the MiBioGen Consortium employed 16S rRNA sequencing technology, which has limited microbial resolution; conclusions are applicable only at the class level and do not support strain-level functional inference. Fourth, constrained by limited overlap between T2DM/AD instrumental variables and bacterial class GWAS loci, reverse MR and MVMR were not testable (effective SNPs < 3 after harmonization), and mediation by T2DM could not be assessed in the present framework. MR studies rely on core assumptions such as instrumental variables influence outcomes solely through exposure and are independent of confounders.^[23,24]^ Although this study assessed pleiotropy and heterogeneity through multiple sensitivity analyses, residual confounding and unknown pleiotropy cannot be entirely ruled out. Future research can be further refined in the following areas: expand GWAS samples across different ethnic groups and sequencing platforms to enhance the ability to decipher the genetic structure of the gut microbiota; conduct MR analysis at higher-resolution taxonomic levels to identify key genera, species, and their metabolic products; and by integrating longitudinal cohort studies, multi-omics analysis, and animal model experiments, systematically evaluate the biological pathways and translational relevance of Deltaproteobacteria and Clostridia in the development of T2DM and AD. This will provide stronger evidence to inform future mechanistic and interventional study designs targeting the gut ecosystem.

## 5. Conclusion

A 2-sample MR analysis based on pooled large-scale GWAS data revealed evidence consistent with genetically predicted increased *Clostridia* abundance and reduced risk of AD among 10 predefined gut bacterial classes. Conversely, genetically predicted elevated *Deltaproteobacteria* abundance was associated with increased risk of T2DM. However, no robust evidence for a direct genetically proxied effect of T2DM on AD was observed. This study provides novel evidence from a genetic epidemiological perspective for the connection between gut microbiota, metabolic disorders, and neurodegenerative diseases. Given the predominantly European-ancestry GWAS sources, limited microbiome instrument strength, and class-level resolution, these findings should be viewed as hypothesis-generating and require replication in diverse ancestries and follow-up mechanistic/experimental and interventional designs to clarify mechanisms and translational relevance.

## Author contributions

**Conceptualization:** Xueying Gao, Lingling Wang.

**Data curation:** Xueying Gao, Lingling Wang.

**Formal analysis:** Lingling Wang.

**Software:** Lingling Wang.

**Writing – original draft:** Xueying Gao, Lingling Wang.

**Writing – review & editing:** Xueying Gao, Lingling Wang.




