# Supplementary material for: Associations of gut bacterial classes Clostridia and Deltaproteobacteria with type 2 diabetes and Alzheimer’s disease: A two-sample Mendelian randomization study
Source: Medicine (Baltimore). 2026 May 15;105(20):e48685. doi: 10.1097/MD.0000000000048685 (PMC13183148; doi:10.1097/MD.0000000000048685)
Supplement: Supplementary file 1 [file medi-105-e48685-s001.doc]

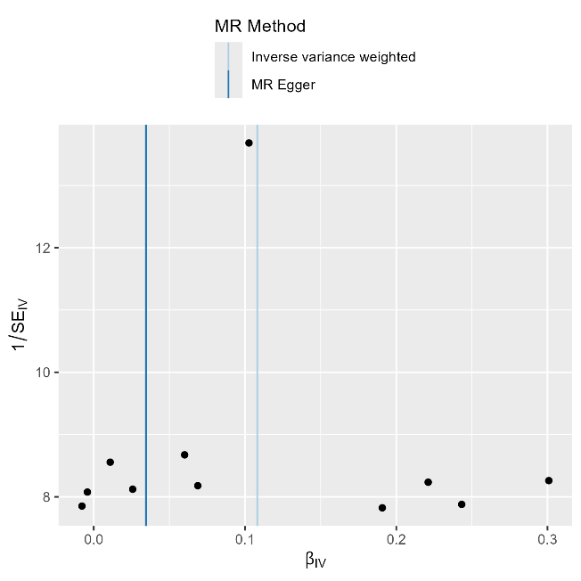


**Supplemental Digital Content 1.** Sensitivity analyses for the MR association between Deltaproteobacteria and Type 2 Diabetes Mellitus. **(A)** Funnel plot displaying the symmetrical distribution of SNPs, suggesting an absence of significant directional pleiotropy. The vertical lines represent the MR estimate.


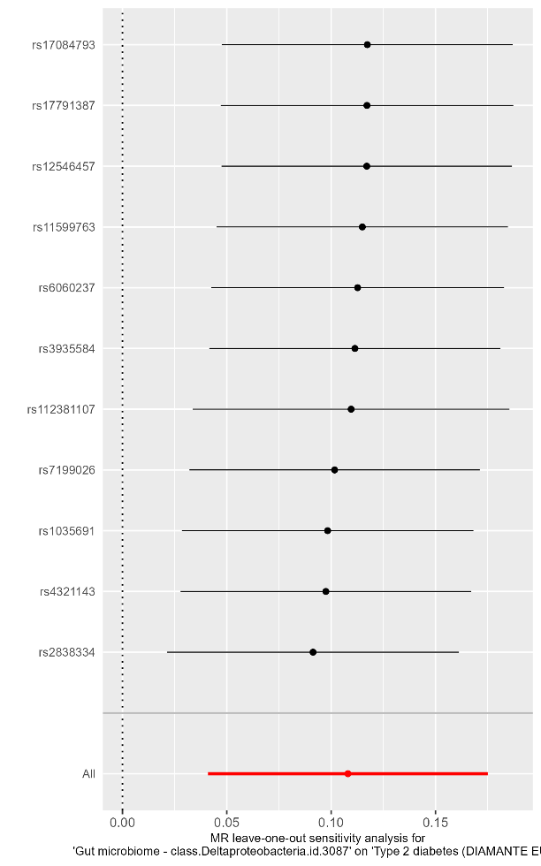


**Supplementary Figure 1** Sensitivity analyses for the MR association between Deltaproteobacteria and Type 2 Diabetes Mellitus. **(B)** Leave-one-out sensitivity analysis demonstrating that no single SNP disproportionately drove the overall MR estimate, indicating the stability of the MR estimate.
