# Supplementary material for: Associations of gut bacterial classes Clostridia and Deltaproteobacteria with type 2 diabetes and Alzheimer’s disease: A two-sample Mendelian randomization study
Source: Medicine (Baltimore). 2026 May 15;105(20):e48685. doi: 10.1097/MD.0000000000048685 (PMC13183148; doi:10.1097/MD.0000000000048685)
Supplement: Supplementary file 2 [file medi-105-e48685-s002.doc]

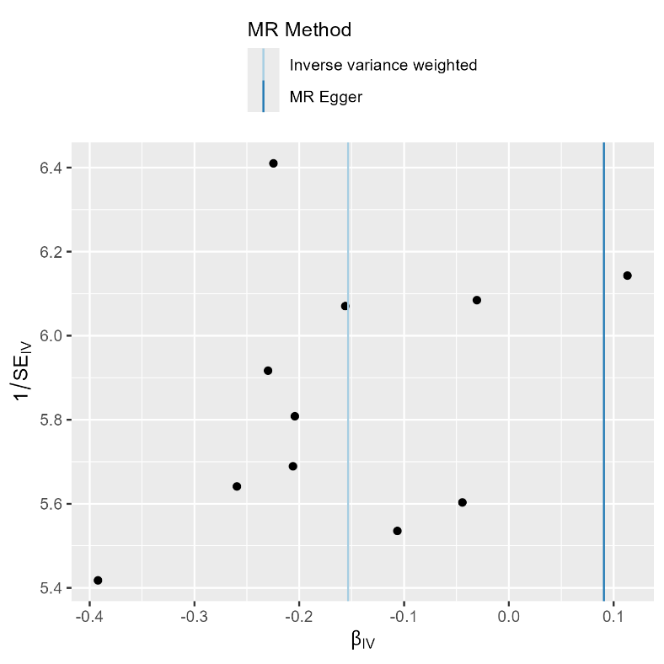


**Supplementary Figure 2.** Sensitivity analyses for the MR association between Clostridia and Alzheimer’s disease. **(A)** Funnel plot assessing potential heterogeneity and directional pleiotropy.


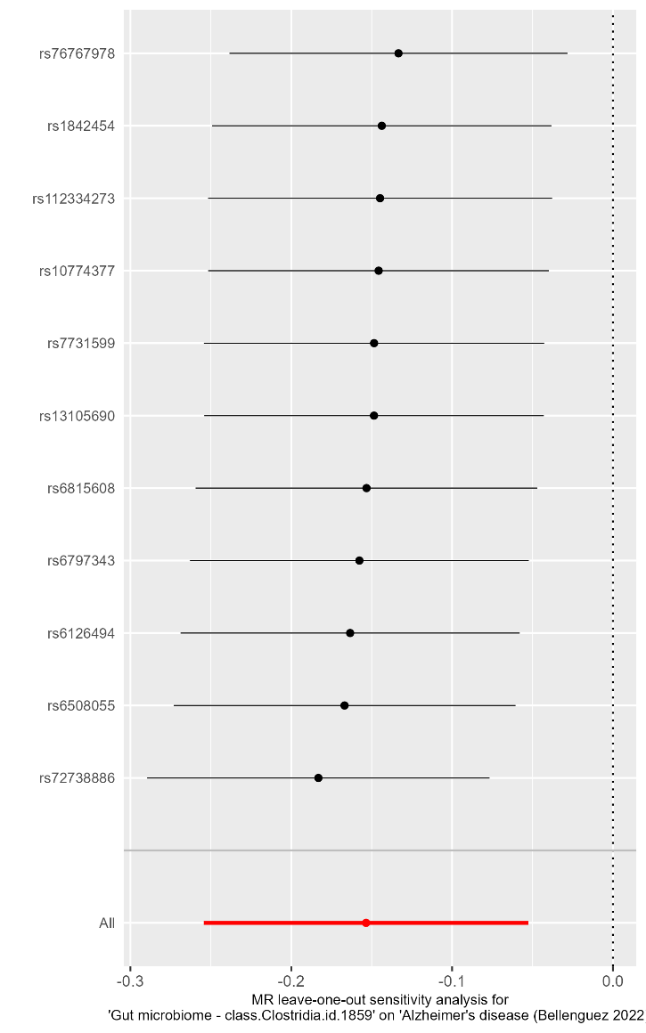


**Supplementary Figure 2.** Sensitivity analyses for the MR association between Clostridia and Alzheimer’s disease. **(B)** Leave-one-out sensitivity analysis verifying the stability of the MR estimate by systematically removing one SNP at a time. The error bars indicate 95% confidence intervals.
